# A systematic review of the effects of Chronic Kidney Disease on lower extremity Peripheral Arterial Disease and Peripheral Neuropathy with recommendations on the development of clinical guidelines

**DOI:** 10.1186/1757-1146-3-S1-P14

**Published:** 2010-12-20

**Authors:** Leigh Porter, Wendy Smith

**Affiliations:** 1 Glasgow Caledonian University, Glasgow, UK

## Objectives and relationship to conference themes

The effects of systemic disease on the lower extremity of high-risk patient groups, namely Diabetes and Rheumatic disease is well recognised and established within the Podiatrist’s scope of practice. However, it appears limited information is known within in the Podiatry profession about patients with Chronic Kidney Disease (CKD) and the onset of lower extremity complications. The main objective of the study was to systematically review the prevalence of lower extremity Peripheral Arterial Disease (PAD) and Peripheral Neuropathy (PN) in CKD and provide recommendations on the development of clinical guidelines.

## Content of presentation

### Introduction

The role of the Podiatrist is to identify the factors which may place the individual’s lower extremity ‘at-risk’. In many cases, these factors do not present in isolation and often co-exist to further increase the risk of the limb. The presence of lower extremity PAD and PN in the development of foot ulceration has been extensively studied in systemically high-risk groups. However, very little evidence appears to be available to support the effects of co-existing risk factors on the lower extremity of individuals with CKD.

### Methods

A systematic review was performed in accordance with the methodology employed within in the Scottish Intercollegiate Guideline Network (SIGN) guideline development process.

### Results

The findings confirmed an increased prevalence of PAD and PN within the CKD population. Prevalence rates of PAD ranged from 19-25.5% and PN from 44-74%. Indeed, placing this patient group at risk for further complications, which in turn increases the risks of lower extremity ulceration and possible amputation.

## Relevance/Impact of Topic

This systematic review has highlighted that the independent presence of PAD and PN in CKD can have profound effects on the viability of the lower extremity. However guidelines, treatment strategies and screening programmes are currently minimal to support this high-risk patient group.

## Participants Outcomes

National frameworks such as SIGN and NICE have recognised the benefits of foot screening programmes to be invaluable in preventing foot related complications within other high-risk groups, such as Diabetes, however this has not included the CKD population. This systematic review would recommend all patients presenting with CKD should be screened for lower extremity vascular and neurological impairment. Furthermore, National guidelines should be developed to highlight the profound effect these risk factors can have on the lower extremity in CKD and encourage the Health Care Professional to undertake simple non-invasive tests to detect and prevent the development of these potentially serious complications.

